# Effects of New NSAID-CAI Hybrid Compounds in Inflammation and Lung Fibrosis

**DOI:** 10.3390/biom10091307

**Published:** 2020-09-10

**Authors:** Laura Lucarini, Mariaconcetta Durante, Silvia Sgambellone, Cecilia Lanzi, Elisabetta Bigagli, Ozlem Akgul, Emanuela Masini, Claudiu T. Supuran, Fabrizio Carta

**Affiliations:** 1 Department of Neuroscience, Psychology, Drug Research and Child Health (NEUROFARBA), Pharmacology and Toxicology Section, University of Florence, Viale G. Pieraccini n. 6, 50139 Florence, Italy; mariaconcetta.durante@unifi.it (M.D.); silvia.sgambellone@unifi.it (S.S.); cecilia.lanzi@unifi.it (C.L.); elisabetta.bigagli@unifi.it (E.B.); emanuela.masini@unifi.it (E.M.); 2 Faculty of Pharmacy, Department of Pharmaceutical Chemistry, Ege University Bornova, 35100 Izmir, Turkey; ozlem.akgul@ege.edu.tr; 3 Department of NEUROFARBA, Pharmaceutical Science Section, University of Florence, Via Ugo Schiff 6, Sesto Fiorentino, 50019 Florence, Italy; claudiu.supuran@unifi.it (C.T.S.); fabrizio.carta@unifi.it (F.C.)

**Keywords:** NSAIDs, CAI, inflammation, pulmonary fibrosis, COX-1, COX-2

## Abstract

Pulmonary fibrosis is a severe lung disease with progressive worsening of dyspnea, characterized by chronic inflammation and remodeling of lung parenchyma. Carbonic anhydrases are a family of zinc-metallo-enzymes that catalyze the reversible interconversion of carbon-dioxide and water to bicarbonate and protons. Carbonic Anhydrase Inhibitor (CAI) exhibited anti-inflammatory effects in animals with permanent-middle-cerebral artery occlusion, arthritis and neuropathic pain. The pharmacological profile of a new class of hybrid compounds constituted by a CAI connected to a Nonsteroidal-Anti-Inflammatory Drug (NSAID) was studied in the modulation of inflammation and fibrosis. In-vitro tests were performed to assess their effects on cyclo-oxygenase enzyme (COX)-1 and COX-2, namely inhibition of platelet aggregation and thromboxane B2 production in the human-platelet-rich plasma, and reduction of Prostaglandin-E2 production in lipopolysaccharide-treated-RAW-264.7 macrophage cell line. The activity of compound **3**, one of the most active, was studied in a model of bleomycin-induced lung fibrosis in C57BL/6 mice. The hybrid compounds showed a higher potency in inhibiting PGE_2_ production, but not in modifying the platelet aggregation and the TXB_2_ production in comparison to the reference molecules, indicating an increased activity in COX-2 inhibition. In the in-vivo murine model, the compound **3** was more effective in decreasing inflammation, lung stiffness and oxidative stress in comparison to the reference drugs given alone or in association. In conclusion, these CAI-NSAID hybrid compounds are promising new anti-inflammatory drugs for the treatment of lung chronic inflammatory diseases.

## 1. Introduction

Pulmonary fibrosis (PF) is defined as specific form of progressive and chronic interstitial pneumonia with unknown etiology, characterized by progressive worsening of dyspnea and function of the lungs and it is characterized by a poor prognosis [1,2]. Nintedanib and pirfenidone have been recently approved as safe drugs for idiopathic pulmonary fibrosis (IPF) treatment by the European Medicines Agency and the Food and Drug Administration (FDA) [3,4]. However, new therapeutic strategies are needed as alternative treatments when the available therapies are not effective or satisfactory [5,6]. In this context are our efforts in finding new and promising therapeutic approaches for the management of such disease. Here we explored the pharmacological profile on a pulmonary fibrosis animal model of a new class of hybrid compounds, recently reported by some of us, constituted by a Carbonic Anhydrase Inhibitor connected to a Nonsteroidal Anti-Inflammatory Drug (CAI-NSAIDs) [7,8].

The α-Carbonic Anhydrase (CA EC 4.2.1.1) family includes 16 catalytically active zinc metallo-enzymes involved in the reversible hydration of carbon dioxide to afford bicarbonate and a proton as products. The enzymatic CA reaction is involved in many pathological and physiological processes, including pH and CO_2_ homeostasis, electrolyte secretion, bone resorption, and tumorigenicity [9]. The isoforms differ mainly in their subcellular localization, tissue distribution and catalytic activity [10]. Inhibitors of the CAs are in clinical use for the treatment of intraocular hypertension in glaucomatous patients [11], epilepsy [10], edema, and promising candidates for the management of obesity [9]. Advanced clinical investigations are currently ongoing on cancers, typically the hypoxic ones, overexpressing the CA IX isoform [12]. Recently, a relationship between hypoxia, CAs IX and XII over-expression, and ischemia has been highlighted [13]. Such a concept was proved by means of Carbonic Anhydrase Inhibitors (CAIs) of the sulfonamide type which exhibited anti-inflammatory effects in rats with permanent middle cerebral artery occlusion (pMCAO) [13]. The neurological score of pMCAO in rats was dramatically reduced 24 h after occlusion and the properties of novel CAIs to improve the neurological functionalities after cerebral ischemic insult are shown [13].

Cyclo-oxygenase enzymes (COXs) catalyze the transformation of arachidonic acid into prostaglandin-H_2_ (PGH_2_), which constitutes the first step in the biosynthesis of prostanoids. To date, two COX enzymes (COX-1 and COX-2) are reported and both catalyze the same reaction. COX-1 is constitutively expressed in human tissues and it is involved in physiological functions such as mucous production and renal excretion through the synthesis of prostaglandins and platelet aggregation by means of thromboxane A_2_ (TXA_2_). This isoform is responsible for producing prostaglandins that activate platelets and it is involved in the protection of the stomach and intestinal lining from the gastric acids. PGH_2_ derived from COX-1 plays important roles in homeostatic processes such as thrombo-genesis and homeostasis of the gastrointestinal tract and kidney [14]. COX-2 is an inducible isoform overexpressed when inflammation occurs. COX-2 expression is also observed in some tissues such as vascular endothelium, kidney or brain under normal conditions, thus suggesting the involvement of COX-2 in the regulation of physiological processes too [15].

**Figure 1:**
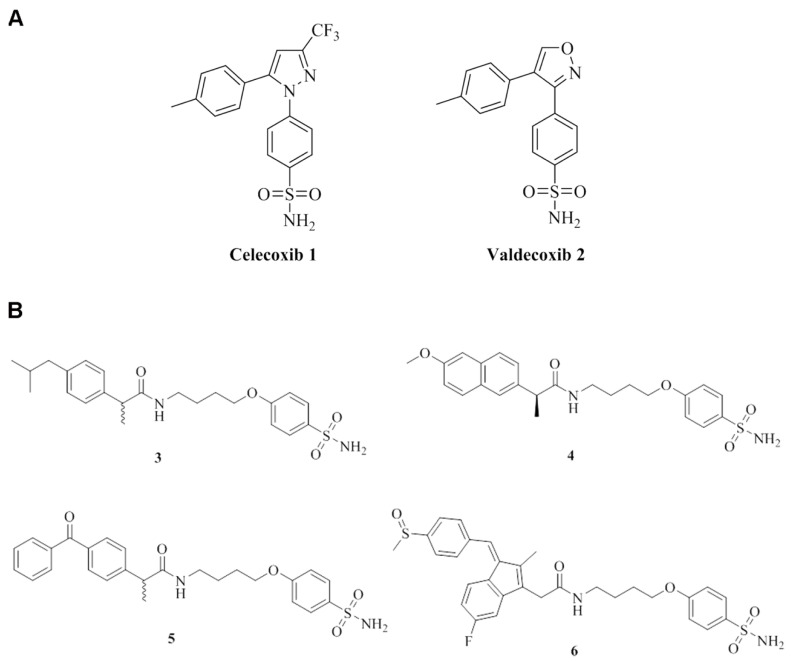
Chemical structures. (**A**) celecoxib 1 and valdecoxib 2. (**B**) chemical structures and molecular weights of the studied compounds **3**–**6** [7].

Among the class of NSAIDs, currently used, are the COX-2 inhibitors. Some of us reported an in vitro kinetic investigation of COXIBs possessing the canonical CA inhibitory moiety (i.e., the primary sulfonamide group) for their ability to inhibit the CAs (Figure 1A).

Quite interestingly, the COXIB 1 and 2 were quite effective inhibitors of the tumor-associated hCA IX isoform with K_I_ values within the nanomolar range [7,8]. The dual inhibition of CA IX and COX-2 enzymes reported in such a study, further sustained the use of a poly-pharmacological approach for cancer treatment [16]. It should be stressed that derivatives showing COX-2 selective inhibition profiles could be preferred since the side effects related to the inhibition ubiquitous COX-1 isoform are suppressed.

The scientific contributions discussed above on the use of a dual targeting strategy for the management of diseases [7,8] represent the blueprint on which the current study stands on. We performed in vitro tests to assess the effects of new CAI-NSAID selected compounds on the arachidonic acid cascade in inflammation processes, namely platelet aggregation and thromboxane B_2_ production in human platelet-rich plasma to evaluate the COX-1 (ubiquitous) inhibition and the reduction of PGE_2_ production in lipopolysaccharide (LPS)-treated macrophage cell line to appraise the COX-2 (over-expressed in inflammation) inhibition. Moreover, the activity of one of the most active molecules was subsequently studied in a murine model of bleomycin-induced lung fibrosis, in comparison to the reference drugs.

## 2. Materials and Methods

### 2.1. Drugs and Reagents

The studied compounds **3**–**6** were synthesized according to the previously reported chemical procedures [7], and their chemical structures, are depicted in Figure 1B.

The compounds selected in this study were all characterized by the primary sulfonamide CAI head section spaced through a *O*-*n*-butyl chain to the appropriate NSAID terminals such as the (±)-ibuprofen, *S*-(+)-naproxen, (±)-ketoprofen and sulindac for **3** to **6** respectively. In order to properly address a suitable chemical stability to the final CAI-NSAIDs we considered the amidic functionality which was readily installed by ordinary coupling reactions between the carboxylic acid of the NSAIDs and the primary amine of the CAI bearing spacer [7].

Compounds **1**–**6** were used in a model of inflammation with murine macrophage RAW 264.7 cell line to measure the production of PGE_2_ in order to evaluate their activity on COX-2 enzyme and in human platelet-rich plasma activated with adenosine diphosphate (ADP) to measure the inhibition of platelet aggregation and of thromboxane B_2_ production, in order to evaluate their activity on COX-1 enzyme. These compounds were used at the following concentrations: 100 nM, 1 µM, 10 µM and 100 µM dissolved in 1% DMSO in PBS buffer.

NSAIDs ((±)-ibuprofen, (*S*)-(+)-naproxen, (±)-ketoprofen and sulindac) and acetazolamide (AAZ) were purchased from a commercial dealer (Sigma-Aldrich, Milan, Italy), and they were used at a concentration of 1 µM and/or 100 µM dissolved in vehicle (1% DMSO in PBS buffer) in the model of inflammation with macrophage cell line and in human platelet-rich plasma.

In the in vivo model of bleomycin-induced lung fibrosis, the (±)-ibuprofen-sulfonamide derivative **3**, was used at a concentration of 1 mg/kg b.wt in the bleomycin-induced model of lung fibrosis, and it was dissolved in 1% DMSO in PBS buffer. We selected this compound because ibuprofen is a widely used COX-1/COX-2 inhibitor and it had a good bioavailability for micro-pumps’ administration.

The (±)-ibuprofen and AAZ were used as reference drugs and they were administered alone or in co-administration at a concentration of 0.5 mg/kg b.wt.

Bleomycin (Merck-Millipore, Milan, Italy) was used at a concentration of 0.05 IU for each mouse and dissolved in 50 μL of PBS buffer, in order to obtain the bleomycin-induced model of lung fibrosis.

### 2.2. Cell Culture Conditions

The murine macrophage cell line RAW 264.7 was cultured in Dulbecco’s modified Eagle’s medium (DMEM) supplemented with 10% fetal bovine serum (FBS) and 1% penicillin (10,000 U/mL)-streptomycin (10,000 μg/mL) at 37 °C in a humidified incubator containing 5% CO_2_. Cells were left to adapt for 24 h before any treatments in all experiments.

#### 2.2.1. Lipopolysaccharide (LPS) Induced Inflammation in RAW 264.7

RAW 264.7 macrophage cells, seeded at a concentration of 2 × 10^4^ cells/well, were cultivated on 12-well plates and treated with 100 nM, 1 µM, 10 µM and 100 µM of each compound. Cells were pretreated (1 h before) with 1 μg/mL LPS to induce inflammation [17]. Eighteen hours later, at the end of the treatment, cell-free culture media were harvested. Cell lysates were collected and stored at −80 °C for further experiments.

#### 2.2.2. Determination of Prostaglandin E_2_ (PGE_2_) Levels

The concentrations of PGE_2_ in the culture media of RAW 264.7 cells were determined using Prostaglandin E_2_ ELISA kit (Cayman Chemical, Ann Arbor, MI, USA), according to the manufacturer’s instructions.

#### 2.2.3. Human Platelet-Rich Plasma

Human platelet-rich plasma (PRP) was obtained from human whole blood from male healthy donors, who signed an informed consent. None of them took any COX inhibitor or any other drugs within 20 days before entering the study. Blood is drawn with the addition of the anticoagulant citrate dextrose A (1:10 *v*/*v*) to prevent platelet activation prior to use.

PRP was obtained by centrifugation of the whole blood at 250× *g* for 15 min at 20 °C. Samples were used for the aggregation studies after adjustment of platelet count to 3 × l0^8^ per ml with platelet-poor plasma [18].

#### 2.2.4. Measurement of Platelet Aggregation (PA)

Turbidimetric PA was used to measure agonist-induced aggregation using an APACT 4 aggregometer (Helena Laboratories Italia S.P.A, Milan, Italy). PRP was stimulated with 10 μM ADP (Menarini s.r.l. Florence, Italy). Platelet aggregation was evaluated, according to Born’s method, considering the maximal percentage of platelet aggregation in response to stimulus (ADP-PA) after 5 min, both in presence and in absence of the studied compounds [18].

#### 2.2.5. Thromboxane B_2_ (TXB_2_) Production

The concentration of TXA_2_ was measured as TXB_2_ production with an ELISA method on PRP after clotting. By measuring TXB_2_, a stabilized metabolite of TXA_2_, the pharmacodynamic efficacy of our compounds was studied in comparison to the reference molecule of origin. TXB_2_ was measured using the Thromboxane B_2_ Express EIA kit – Monoclonal (Cayman Chemical, Ann Arbor, MI, USA), according to the manufacturer’s instructions.

### 2.3. Animals

The animals were fed with a standard diet and housed for 5 days under a 12-h light/dark cycle before the experiments. Experiments were carried out at the Centre for Laboratory Animal Housing and Experimentation (CeSAL), University of Florence, Italy. Experiments were performed upon the authorization from the Italian Health Ministry (Authorization N° 874/2017-PR), in agreement with the European Union Regulations (OJ of ECL 358/1, 12/12/1986). Animal care was in accordance with regulations in Italy (DL 26/2014) and Europe (EU Directive 2010/63/EU). The study complies with the ARRIVE guidelines.

#### 2.3.1. Model of Bleomycin-Induced Lung Fibrosis

Bleomycin is a chemotherapeutic agent that is known to cause pulmonary fibrosis as an uncommon side effect in humans undergoing therapy with this agent for cancer [19]. Bleomycin causes oxidative damage to the deoxyribose of thymidylate and other nucleotides leading to single- and double-stranded breaks in DNA, inducing lung injury [20].

Thirty-six C57BL/6 WT mice (~2 months old and weighing 25–30 g) were obtained from a commercial dealer (Envigo, Udine, Italy). They were anesthetized with zolazepam/tiletamine (Zoletil, 50/50 mg/mL, Virbac Srl, Milan, Italy; 50 μg/g i.p. in 100 μL of saline); 30 of them were treated with a single dose of bleomycin(0.05 IU) diluted in 50 μL of PBS, administered by intra-tracheal injection, while 6 mice were treated with 50 μL of PBS buffer (referred to as non-fibrotic negative controls, Naïve), both delivered by intra-tracheal injection [21]. The bleomycin-treated mice, 6 per group, were treated with continuous infusion of drugs by osmotic micropumps (Alzet, Cupertino, CA, USA). Pumps were filled with 100 μL of PBS pH 7.4, containing the drugs at the reported concentrations: compound **3** (1 mg/kg b.wt.), (±)-ibuprofen (Ibu) (0.5 mg/kg b.wt.), AAZ (0.5 mg/kg b.wt.) and Ibu+AAZ co-administration (0.5 mg/kg b.wt. + 0.5 mg/kg b.wt.). Six mice were treated only with PBS buffer and referred to as fibrotic positive controls (Vehicle). The micropumps, that release 1.55 μL of solution per day, were implanted subcutaneously into a dorsal poach at day 0 and maintained for 21 days.

The body weight control of each mouse was carried out once a day for 21 days of treatment in order to exclude any toxicity of the molecules.

#### 2.3.2. Functional Assay of Fibrosis

At day 21 after surgery, the mice were subjected to measurement of airway resistance to inflation, defined as pressure at the airway opening (PAO), a functional parameter related to fibrosis-induced lung stiffness [22]. Briefly, upon anesthesia, a 22-gauge cannula (Venflon 2; Viggo Spectramed, Windlesham, UK, 0.8 mm diameter) was inserted into the trachea and the mouse was ventilated with a small-animal respirator (Ugo Basile, Comerio, Italy), adjusted to deliver a constant volume of 0.8 mL at a rate of 20 strokes/min. Changes in PAO were registered by a high-sensitivity pressure transducer (P75 type 379; Harvard Apparatus Inc., Holliston, MA, USA) connected to a polygraph (Harvard Apparatus Inc. Edenbridge, UK; settings: gain 1, chart speed 25 mm/s). Changes in PAO, measured for at least 3 min and expressed as millimeters, were carried out on at least 40 consecutive tracings of respiratory strokes and then averaged.

#### 2.3.3. Lung Tissue Sampling

After the functional assay, the gross appearance of the lungs was examined, they were excised, and lung wet weight was determined. No macroscopic alterations of this organ or other organs, that could be related to a toxic effect of the treatments were observed. The whole left lungs were excised and fixed by immersion in 4% formaldehyde in PBS for histological analysis. The right lungs were quickly frozen and stored at −80 °C.

At the moment of the biochemical measurements, portions of lung (~20 mg) were thawed at 4 °C, homogenized on ice in 50 mM Tris-HCl buffer containing 180 mM KCl and 10 mM EDTA, pH 7.4, and, then, centrifuged at 10,000× *g*, 4 °C, for 30 min. The supernatants were collected for the following biochemical determinations.

#### 2.3.4. Histology and Assessment of Collagen Deposition and Lung Remodeling

Six-micrometer-thick histological sections were cut from the paraffin-embedded lung samples. All sections were stained in a single session, in order to minimize artefactual alterations in the staining process. Photomicrographs of the histological slides were randomly taken with a digital camera connected to a light microscope equipped with objectives at different magnification. Densitometry was performed to obtain quantitative evaluation of the stained sections. Optical density (OD) and surface area were measured with the free-share ImageJ 1.33 image analysis program. For each measured parameter, values are means ± SEM of the OD measurements (arbitrary units) of individual mouse (five images each) from every experimental groups (tested blind).

The picrosirius red staining was carried out to confirm the assessment of lung collagen; the sections were stained in 0.1% Direct Red 80/Sirius Red F3B (Sigma-Aldrich) in saturated picric acid at room temperature for 1 h, and then they were differentiated in 0.5% acetic acid, prior to dehydration, clearing, and mounting [23,24].

Moreover, lung tissue sections were stained with hematoxylin and eosin or with periodic acid-Schiff (PAS) staining for mucins in order to obtain morphometry of smooth muscle layer thickness and bronchial goblet cell number, respectively, both key markers of airway remodeling. The thickness of the bronchial smooth muscle layer was measured on the digitized images using the above-mentioned software. Total bronchial epithelial cells and PAS-stained goblet cells were counted on bronchial cross-section profiles, and the percentage of goblet cells was calculated.

#### 2.3.5. Determination of Cytokines Production

The levels of two pro-inflammatory cytokines, Interleukin-1β (IL-1β) and Tumor Necrosis Factor-α (TNF-α), and the levels of Transforming Growth Factor-β (TGF-β), the main profibrotic cytokine involved in fibroblast activation, were measured on aliquots (20 μL) of lung homogenate supernatants by using the FlowCytomix assay (Bender Medsystems GmbH, Vienna, Austria), following the protocol provided by the manufacturer. Briefly, suspensions of anti-IL-1β or TNF-α or TGF-β coated beads were incubated with samples and with IL-1β or TNF-α or TGF-β standard curves, and then with biotin-conjugated secondary antibodies and streptavidin-phycoerythrin. Fluorescence was read with a cytofluorimeter (CyFlow^®^ Space, Partec, Carate Brianza, MB, Italia).

The levels of the anti-inflammatory cytokine Interleukin-10 (IL-10) were measured on aliquots (100 μL) of lung homogenate supernatants by using the mouse IL-10 ELISA Ready-SET&Go!^®^ assay (eBioscience, San Diego, CA, USA), following the protocol provided by the manufacturer.

Values are indicated as means ± SEM of 6 individual mice from each group evaluated in duplicate and expressed as pg/μg of total proteins determined over an albumin standard curve.

#### 2.3.6. Determination of Myeloperoxidase (MPO) Activity

Frozen lung samples were weighed and homogenized (10 μL/mg of tissue) in 0.2 M PBS (pH 6), supplemented with protease inhibitors (1 mM PMSF, 20 μg/mL leupeptin, 1 μg/mL pepstatin, 1 mg/mL Pefabloc SC, and 2.5 μg/mL aprotinin, Sigma-Aldrich) and were centrifuged at 10,000× *g* at 4 °C for 30 min. MPO was measured in the supernatants with a specific immunoassay kit (CardioMPO; PrognostiX, Cleveland, OH, USA), according to the manufacturer’s instructions [22]. Total protein concentration in the lung tissue samples was determined over an albumin standard curve. The results are expressed as picomoles/mg of protein. Values are means ± SEM of individual mice from different experimental groups.

#### 2.3.7. Determination of 8-Hydroxy-deoxyGuanosine (8OH-dG)

Lung DNA isolation was performed as previously described [25,26] with minor modifications. In brief, lung samples were homogenized in 1 mL of 10 mM PBS (pH 7.4), sonicated on ice for 1 min, added to 1 mL of 10 mM Tris-HCl buffer (pH 8), containing 10 mM EDTA, 10 mM NaCl, and 0.5% SDS, and incubated for 1 h at 37 °C with 20 μg/mL RNase 1 (Sigma-Aldrich). Samples were incubated at 37 °C overnight with 100 μg/mL proteinase K (Sigma-Aldrich). Chloroform/isoamyl alcohol (10:2, *v*/*v*) was added to the mixture. DNA was separated with 0.2 volumes of 10 M ammonium acetate, dissolved in 200 μL of 20 mM acetate buffer (pH 5.3), and denatured at 90 °C for 3 min. Ten IU of P1 nuclease (Sigma-Aldrich) in 10 μL was added and the solution was incubated for 1 h at 37 °C with 5 IU of alkaline phosphatase (Sigma-Aldrich) in 0.4 M phosphate buffer (pH 8.8). The solution was filtered by an Amicon Micropure-EZ filter (Merck-Millipore), and 100 μL of each sample was used for 8-OHdG determination by using an ELISA kit (JalCA, Shizuoka, Japan), following the instructions provided by the manufacturer. The absorbance of the chromogenic product was measured at 450 nm. The results were calculated from a standard curve based on an 8-OH*d*G solution and expressed as ng of 8-OH*d*G/ng of total DNA.

#### 2.3.8. Fluorescent Dye Dihydroethidium (DHE) Staining

Histological sections, 5-μm-thick, were cut from the paraffin-embedded lung samples, deparaffinized and incubated with fluorescent DHE 2 μM (Abcam, Cambridge, UK) in a humidity chamber for 30 min at 37 °C in the dark. The sections were mounted in an aqueous medium (Flouremont, Sigma, Milan, Italy) and fluorescence was detected (absorbance 518 nm, emission 605 nm) with an Olympus BX61 microscope coupled to CellSens Dimension Imaging Software, version 1.6 (Olympus, Milan, Italy).

### 2.4. Statistical Analysis

Data were reported as mean% or mean values (± SEM) of independent experiments for platelet aggregation, PGE_2_ production and TXB_2_ production analysis (*n* = 8). For each in vivo assay, data were reported as mean values (± SEM) of individual average measures of the different animals per group (*n* = 6). Significance of differences among the groups was assessed by one-way ANOVA followed by Newman–Keuls post hoc test for multiple comparisons. Calculations were made with Prism 5 statistical software (GraphPad Software, Inc., San Diego, CA, USA). The results were considered to be statistically significant when *p* < 0.05.

## 3. Results

### 3.1. Carbonic Anhydrase Inhibitory Potency

All studied CAI-NSAID hybrids **3**–**6** were evaluated for their inhibition against the cytosolic CA I and II and against the membrane-bound CA IX and CA XII isoforms. The clinically used acetazolamide (AAZ) was used as standard drug and the data are reported in Table 1 [7].

The kinetic data reported in Table 1 allowed us to draw the following structure–activity relationships (SARs):

(*i*) The (±)-ibuprofen-CAI derivative **3** resulted in quite an effective inhibitor of the tumor-associated CA isoforms IX and XII (K_I_s of 14.0 and 6.6 nM, respectively) over the cytosolic I and II (K_I_s of 48.3 and 285.0 nM, respectively). In particular, the isoforms I and IX were 5.2- and 1.8-fold more potent, respectively, when compared to the reference AAZ. As for the remaining isoforms II and XII, the compound **3** resulted a weaker inhibitor than the AAZ (i.e., 23.6- and 1.2-fold less potent).

(*ii*) Similar kinetic profile was also observed for the (+)-naproxen-CAI **4** in comparison to AAZ. However, significant differences between compounds **3** and **4** on the CAs I and II were revealed. As reported in Table 1 compound **4** was 1.5-fold less active against the CA I isoform when compared to **3** (K_I_s of 71.4 and 48.3 nM, respectively). Quite interestingly, the introduction in 3 of the (+)-naproxen tail instead (as in 4) resulted in a significant increase in the inhibition potency against the CA II up to 6.1-fold (K_I_s of 285 and 47 nM, respectively). As for the tumor-associated isoforms, the derivative **4** was a slightly less potent inhibitor against the IX, whereas it was quite effective on the XII being up to 1.6-fold more potent when compared to **3** (K_I_s of 4.2 and 6.6 nM, respectively).

(*iii*) The (±)-ketoprofene derivative **5** was the most effective inhibitor against all the considered CA isoforms. As reported in Table 1 the K_I_ values of **5** against the CAs I, II, IX and XII were 20.9, 6.1, 8.0 and 4.1 nM, respectively, and thus far below the AAZ reference values too.

(*iv*) Conversely the introduction of the sulindac NSAID tail to compound **6**, determined a significant increase in the K_I_ values for all CAs (Table 1). Carbonic anhydrase isoforms I and IX resulted in inhibition at concentrations of 93.8 and 22.0 nM, respectively, which are the highest among the compound series **3**–**6**. As for the remaining CA isoforms II and XII, compound **6** was the second least potent inhibitor after the (±)-ibuprofen derivative **3**.

Overall, the CAI-NSAIDs **3**–**6** proved to be quite effective inhibitors of the CAs considered in this study, with affinities comparable to the reference drug AAZ. The only exception was represented by the (±)-ibuprofen-CAI derivative **3**, which showed a high nanomolar inhibition value.

### 3.2. Determination of PGE_2_ Production

The evaluation of the anti-COX-2 effect of the hybrid compounds was performed evaluating the reduction level of its enzymatic product prostaglandin E_2_ (PGE_2_).

**Figure 2:**
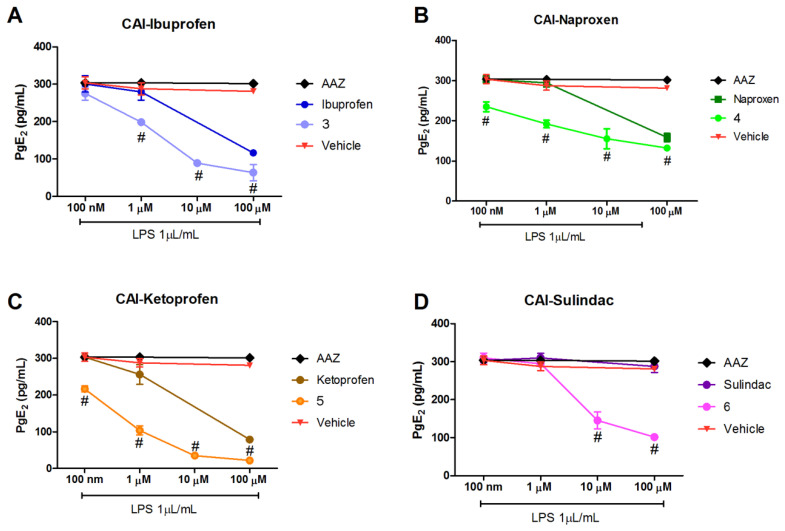
Effects of different concentrations of (**A**) CAI-ibuprofen, (**B**) CAI-naproxen, (**C**) CAI-ketoprofen and (**D**) CAI-sulindac on lipopolysaccharide (LPS)-induced prostaglandin E_2_ (PGE_2_) production in RAW 264.7 cells. The cells were pretreated with the studied compounds (100 nM, 1 µM, 10 µM and 100 µM) for 1 h, followed by LPS (1 μg/mL) stimulation for 18 h. PGE_2_ production analysis was carried out by using cell culture supernatants. PGE_2_ production of Control (naïve cells not treated with LPS) is 36.279 ± 0.67 pg/mL. Values represent the mean ± SEM of eight independent experiments (^#^ *p* < 0.05 between a Nonsteroidal Anti-Inflammatory Drug (NSAID) and a Carbonic Anhydrase Inhibitor connected to a Nonsteroidal Anti-Inflammatory Drug (CAI-NSAID)).

PGE_2_ production was lowered in LPS-stimulated RAW 264.7 macrophages treated with the hybrid compounds with a dose–response effect (Figure 2A–D). All the studied compounds showed efficacy in reducing the production of PGE_2_ as the concentration increases from 100 nM to 100 µM.

Interestingly, all the studied compounds were more effective in reducing PGE_2_ production in comparison to the reference compounds of origin, suggesting an increased activity in inhibiting the COX-2 activation pathway. The CAI-sulindac compound was active in comparison to sulindac itself, which had no effect (Figure 2D). Of note, sulindac is a pro-drug, containing a methyl sulfoxide group, that must be reduced to sulindac sulfide to be active as COX inhibitor [28,29], explaining the non-effect of sulindac alone; while the effect of the hybrid CAI-sulindac is probably due to a different receptor interaction. AAZ, the standard reference as CA inhibitor, was ineffective and very similar to the Vehicle-treated group (Figure 2A–D).

### 3.3. Platelet Aggregation Determination

The evaluation of the anti-COX-1 effect of the hybrid compounds was performed considering the percentage inhibition of platelet aggregation and the level of the eicosanoid thromboxane B_2_ (TXB_2_) production.

**Figure 3:**
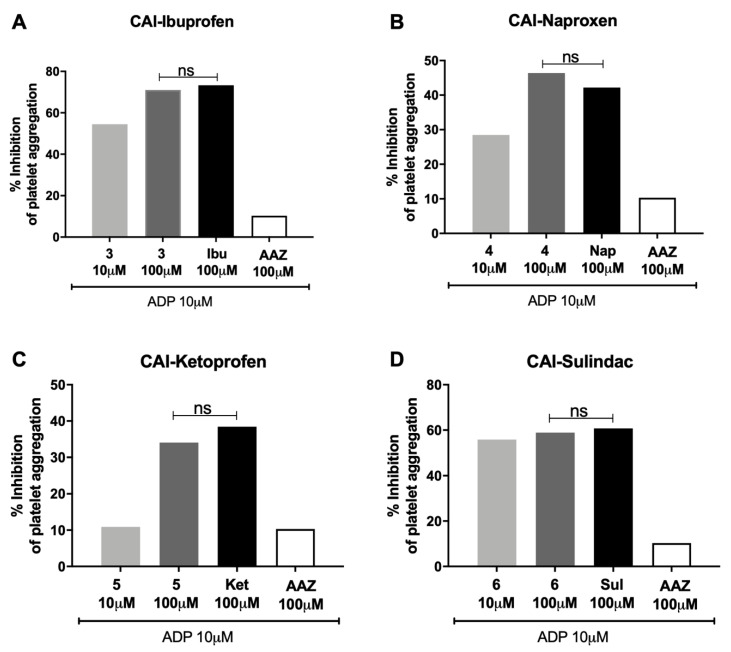
Percentage inhibition of platelet aggregation. Effects of different concentrations of (**A**) CAI-ibuprofen, (**B**) CAI-naproxen, (**C**) CAI-ketoprofen and (**D**) CAI-sulindac after 5 min of ADP (10 µM) incubation in human platelet-rich plasma (PRP). Platelet aggregation baseline is 79.37%, corresponding to 100% of aggregation. Values represent the mean ± SEM of 8 independent experiments, (ns = non-significant between selected columns).

The studied hybrid compounds did not increase the percentage inhibition of platelet aggregation in human PRP in comparison with the reference molecules (±)-ibuprofen (Figure 3A), (*S*)-(+)-naproxen (Figure 3B), (±)-ketoprofen (Figure 3C) and sulindac (Figure 3D), suggesting that these compounds were able to reduce the activity of COX-1 pathway as the starting molecules, but not more than the reference molecules. AAZ, the standard reference as CA inhibitor, was ineffective.

### 3.4. Thromboxane B_2_ Production

**Figure 4:**
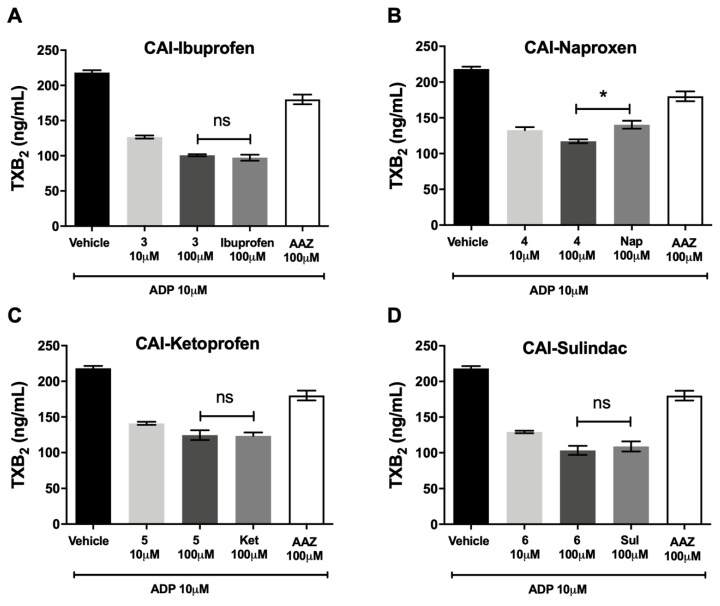
Thromboxane B_2_ production. Effects of different concentrations of (**A**) CAI-ibuprofen, (**B**) CAI-naproxen, (**C**) CAI-ketoprofen and (**D**) CAI-sulindac after 5 min of ADP (10 µM) incubation in human PRP. Values represent the mean ± SEM of eight independent experiments (* *p* < 0.05 between selected columns; ns = non-significant).

CA-IX/COX inhibitors did not decrease the production of TXB_2_, the stable product of TXA_2_, in hPRP, in comparison with the reference molecules (±)-ibuprofen (Figure 4A), (*S*)-(+)-naproxen (Figure 4B), (±)-ketoprofen (Figure 4C)and sulindac (Figure 4D), suggesting that these compounds were not more effective in reducing the activity of COX-1 pathway in comparison with the molecules of origin. AAZ, the standard reference as CA inhibitor, was ineffective.

### 3.5. Functional Assay of Fibrosis

Compound **3** was used as treatment in the bleomycin-induced lung fibrosis model in C57BL/6 mice. (±)-ibuprofen, AAZ and their co-administration were used as reference drugs.

**Figure 5:**
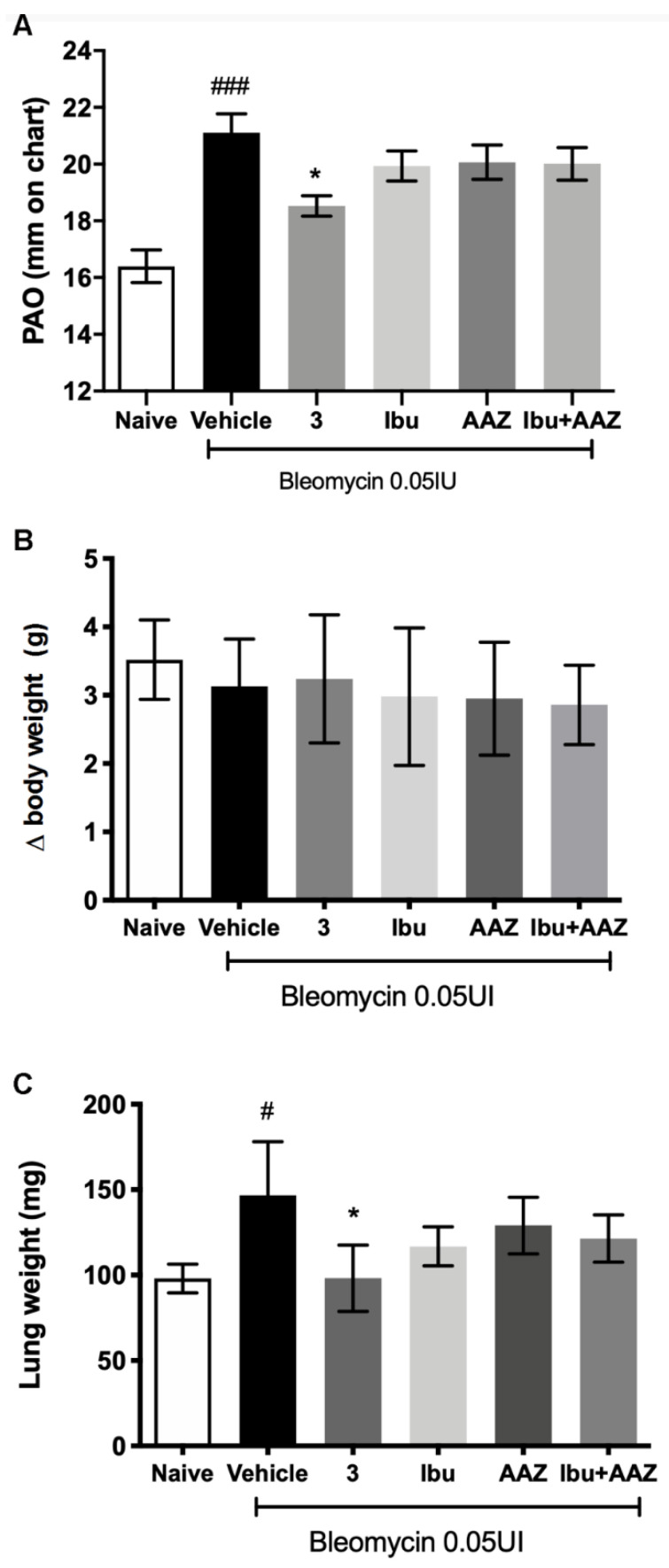
(**A**) Functional measurement of bronchoconstriction. Lung resistance to airflow measured through the evaluation of pressure at airway opening (PAO). Spirometric evaluation (mm on charts) of different treatments. (**B**) Variations of body weight after 3 weeks of treatments; (**C**) measurement of lung weight after bleomycin treatment in presence or absence of various treatments. Values represent the mean ± SEM of 6 individual mice from each group (^###^ *p* < 0.001 vs. Naive; ^#^ *p* < 0.05 vs. Naive; * *p* < 0.05 vs. Vehicle).

Intra-tracheal bleomycin caused a statistically significant increase in airway stiffness, as showed by the clear-cut elevation in the pressure at airway opening (PAO) in the fibrotic positive controls given Vehicle (21.11 ± 0.66 mm) compared with the non-fibrotic negative controls (16.40 ± 0.58 mm). Compounds **3** caused a statistically significant reduction of airway stiffness (18.53 ± 0.36 mm). No significant effects were shown with (±)-ibuprofen (19.94 ± 0.53 mm), AAZ (20.07 ± 0.61 mm) and their co-administration (20.01 ± 0.57 mm). The results of the functional assay are reported in Figure 5A.

The body weight control of each mouse was carried out once a day for 21 days of treatment in order to exclude any toxicity of the molecules. The variation among the groups showed that the treatments did not influence the body weight and in particular compound **3** did not show any toxicity (Figure 5B).

At the end of the functional assay of fibrosis, the gross appearance of the lungs was examined. No macroscopic alterations of this organ or other organs, that could be related to a toxic effect of the treatments were observed. Moreover, lung wet weight was determined; the results showed that lung weight of the Vehicle group was statistically increased in comparison to Naïve group, as is always observed, since a fibrotic lung weighs more in comparison to a physiological one. The treatment with compound **3** reduced significantly the weight of the lungs in comparison to Vehicle treatment (Figure 5C).

### 3.6. Lung Histology

**Figure 6:**
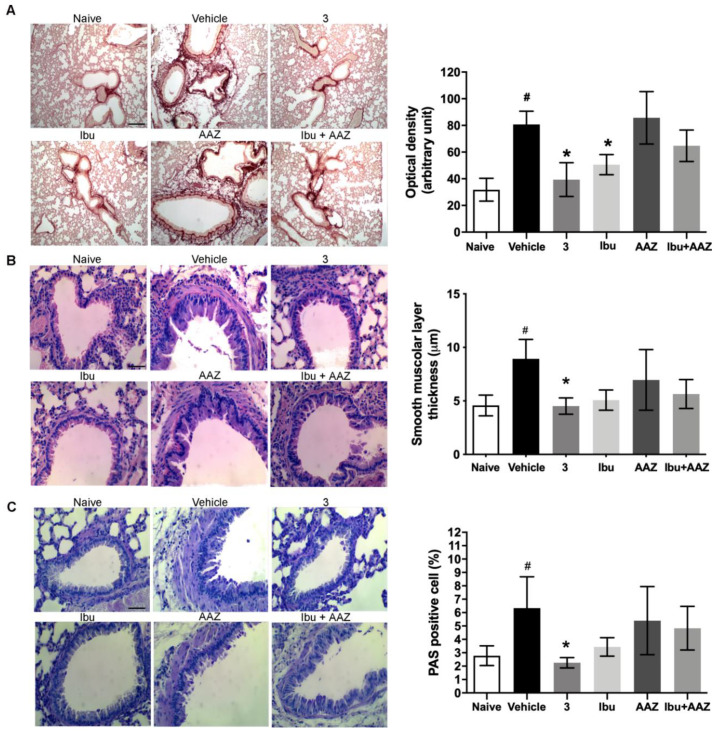
(**A**) Lung histology of collagen deposition. Histopathological evaluation of fibrosis by picro-sirius staining. By computer-aided densitometry analysis, it is possible to obtain a semiquantitative measure of this accumulation. *n* = 6 animals/group. Data are mean ± SEM, ^#^ *p* < 0.05 vs. Naive, * *p* < 0.05 vs. Vehicle. Scale bar, 100 µm (**B**) Histopathological evaluation of airway remodeling by hematoxylin-eosin staining. *n* = 6 animals/group. Data are mean ± SEM. ^#^ *p* < 0.05 vs. Naive, * *p* < 0.05 vs. Vehicle. Scale bar, 50 µm (**C**) Goblet cell number in PAS-stained lung sections (see the arrows) is evaluated in each experimental group. *n* = 6 animals/group. Data are mean ± SEM. ^#^ *p* < 0.05 vs. Naive, * *p* < 0.05 vs. Vehicle. Scale bar, 50 µm.

Morphological observation and computer-aided densitometry on picro-sirius stained sections, which allows the determination of the optical density (OD) of collagen fibers, revealed a significant increase in collagen deposition in the lungs of the bleomycin-treated animals (Vehicle) compared with the non-fibrotic negative controls (Naïve). Treatments with compound **3** and ibuprofen caused a significant reduction of the amount of lung collagen fibers (Figure 6A). No effects were shown with AAZ and the co-administration of AAZ and ibuprofen.

Bronchial remodeling was evaluated by measuring the thickness of the smooth muscle layer (Figure 6B), and the relative number of PAS-positive goblet cells (Figure 6C), key histological parameters of inflammation-induced adverse bronchial remodeling. As expected, both these parameters significantly increased in the bleomycin-treated mice. Compound **3** significantly reduced the thickness of the airway smooth muscle layer, as well as the percentage of PAS-positive goblet cells versus total bronchial epithelial cells. Ibuprofen was shown to be slightly less effective and AAZ was totally ineffective, while the co-administration of these compounds had a slight effect.

### 3.7. Determination of Pro- and Anti-Inflammatory Cytokines

**Figure 7:**
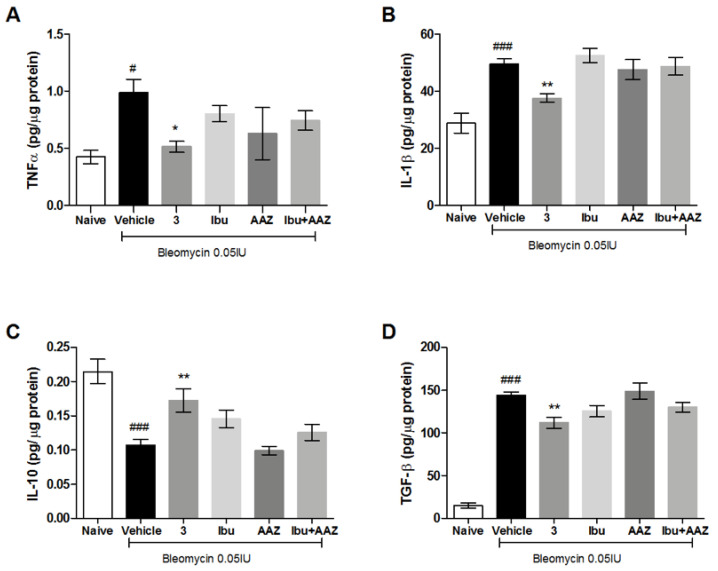
Determination of pro-inflammatory (Interleukin-1β (IL-1 β) and Tumor Necrosis Factor-α (TNFα)), anti-inflammatory (IL-10) and pro-fibrotic (Transforming Growth Factor-β (TGF-β)) cytokines. Analysis of (**A**) IL-1 β, (**B**) TNFα, (**C**) IL-10 and (**D**) TGF-β content in the supernatant of lung tissue homogenates. Values represent the mean ± SEM of six individual mice from each group performed in duplicate and expressed as pg/μg of total proteins determined over an albumin standard curve. ^#^ *p* < 0.05 and ^##^ *p* < 0.005 vs. Naive; ^###^ *p* < 0.001 vs. Naive; * *p* < 0.05 vs. Vehicle; ** *p* < 0.005 vs. Vehicle.

The late (21 days after challenge) inflammatory response to bleomycin was evaluated by measuring the cytokines IL-1β and TNF-α in the supernatant of lung homogenates. Bleomycin treatment increased IL-1β and TNF-α production in the lungs, while the systemic administration of the compound **3** significantly decreased the levels of IL-1β and TNF-α cytokine production. The treatment with (±)-ibuprofen and AAZ was not effective in the reduction of inflammatory cytokine production, as well as in their co-administration (Figure 7A,B).

In order to confirm the anti-inflammatory activity of the compound **3**, we evaluated the levels of IL-10, the most potent anti-inflammatory cytokine involved in resolution of different acute and chronic inflammatory diseases [30]. Our results reported a significant increase in the production of IL-10 in mice treated with compound **3** in comparison to mice treated with Vehicle or AAZ, while the treatment with (±)-ibuprofen, as well as the co-administration of (±)-ibuprofen and AAZ, did not significantly modified the IL-10 production (Figure 7C).

Overall, these data indicate that compound **3** attenuated the bleomycin-induced inflammatory responses and suggest a significant synergistic effect of the hybrid compound in comparison to the combination of single drugs, which states the efficacy of the adopted multi-target strategy.

### 3.8. Determination of TGF-β Pro-Fibrotic Cytokine

TGF-β, an important pro-fibrotic cytokine, is increased in the Vehicle group, suggesting that an intensified TGF-β signaling contributes to the development of pulmonary fibrosis.

Systemic administration of compound **3** causes a significant decrease in TGF-β production, while the treatments with ibuprofen, AAZ and their co-administration do not decrease significantly the production of this cytokine (Figure 7D). These results demonstrate the anti-fibrotic activity of compound **3**.

### 3.9. Determination of Leukocyte Lung Infiltration and of Oxidative Stress Marker

Myeloperoxidase (MPO) is a peroxidase enzyme abundantly expressed in neutrophils and monocytes/macrophages granules, and it is considered a reliable marker for leukocyte accumulation in inflamed tissues [31].

**Figure 8:**
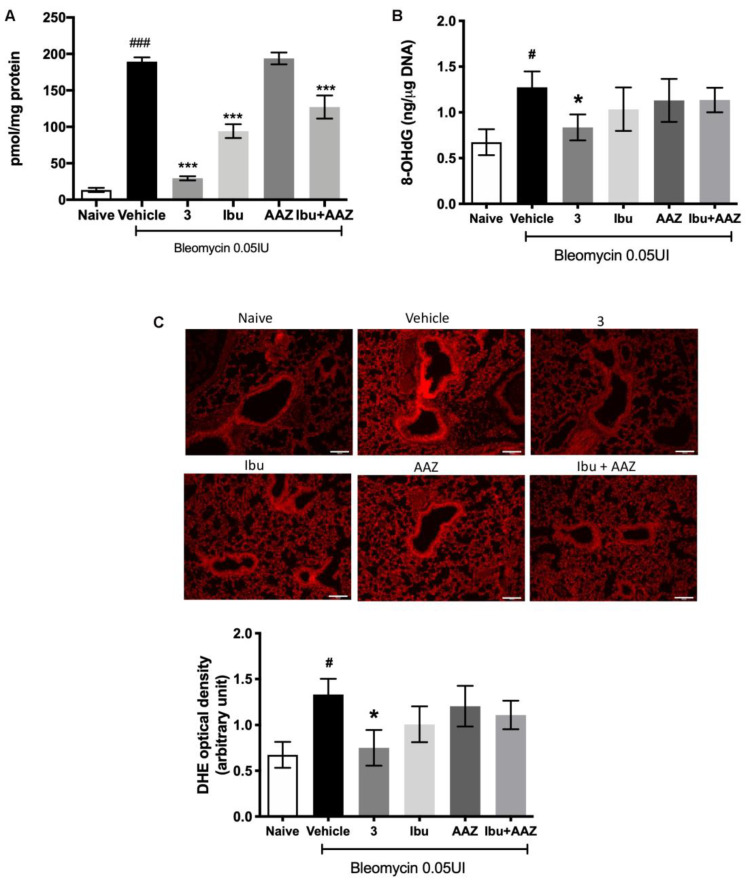
Evaluation of oxidative stress parameter in lung tissue. (**A**) Levels of myeloperoxidase (MPO), a marker for leukocyte accumulation in inflamed tissues. (**B**) Levels of 8-OH*d*G, a marker of free radicals-induced DNA damage; (**C**) dihydroethidium (DHE), a morphological marker of DNA damage and the densitometer evaluation. *n* = 6 animals/group. Values are mean ± SEM. ^#^ *p* < 0.05 vs. Naive. ^###^ *p* < 0.001 vs. Naive. * *p* < 0.05 vs. Vehicle. *** *p* < 0.001 vs. Vehicle. Scale bar, 100 µm.

Levels of lung MPO were very low in the Naïve mice and significantly increased in the bleomycin-treated mice (Vehicle group). A significant decrease in MPO production was demonstrated after treatment with compound **3**, ibuprofen and the co-administration of ibuprofen and AAZ in comparison to Vehicle; acetazolamide alone has no effects (Figure 8A).

Oxidative stress was quantitatively evaluated by determining 8OH*d*G, a biological marker of DNA damage and, morphologically with DHE staining.

The determination of 8-OH*d*G demonstrates that it is significantly increased in bleomycin-exposed animals (Vehicle), compared with non-fibrotic negative ones (Naïve). Interestingly, a significant reduction of 8-OH*d*G levels in animals treated with compound **3** was observed, but not with ibuprofen, acetazolamide, or their co-administration (Figure 8B). DHE staining demonstrated that the intensity of signal was very strong in the lungs of Vehicle-treated animals in comparison with the control group (Naïve). Treatment with compound **3** reduced significantly the signal intensity, thus meaning less damage to DNA, while treatments with ibuprofen, AAZ and their co-administration had no significant effects (Figure 8C).

## 4. Discussion

A new class of hybrid compounds endowed with anti-inflammatory and carbonic anhydrase IX inhibition activities have been investigated in this study to evaluate their anti-inflammatory activity. These compounds have been studied in vitro in a macrophage cell line and in human platelet-rich plasma (hPRP) to select their inhibitory activity on COX-1 and COX-2. Moreover, the (±)-ibuprofen derivative hybrid compound **3**, the most active molecule among those studied, was then tested in an in vivo mouse model of bleomycin-induced lung fibrosis.

When looking at the reduction of PGE_2_ production in LPS-stimulated murine macrophage RAW 264.7 cells, which depends mostly on COX-2 inhibition, it is very interesting to note that the activity of the new hybrid NSAID/CAI compounds were increased in comparison to the reference drugs; in fact, (±)-ibuprofen, (*S*)-(+)-naproxen and (±)-ketoprofen at 100 μM were less effective in decreasing the production of PGE_2_, than the hybrid compounds; sulindac and AAZ (100 μM) were ineffective. Therefore, these compounds might slow down the progression of disease states resulting from chronic inflammation by regulating inflammatory prostaglandin production.

On the other hand, these molecules did not increase the percentage inhibition of platelet aggregation in hPRP in comparison with the reference molecules (±)-ibuprofen, (*S*)-(+)-naproxen, (±)-ketoprofen and sulindac, and they did not decrease the production of TXB_2_, the stable product of TXA_2_, suggesting that these compounds are not more effective in reducing the activity of COX-1 pathway in comparison with the molecules of origin; of note, acetazolamide was ineffective.

The anti-inflammatory and anti-fibrotic properties of the (±)-ibuprofen derivative hybrid molecule (compound **3**) in a mouse model of lung fibrosis was further investigated. We selected the model of bleomycin, intra-tracheally delivered, because it is the best characterized murine model in use today for lung fibrosis and, nowadays, there are no approved drugs that counteract the pathological mechanism of this disease, apart some potential treatments targeting the TGF-β pathway, pirfenidone, and the tyrosine kinase inhibitor, nintedanib [32,33,34].

Our experiments were performed in C57BL/6 mice, which are more susceptible to bleomycin-induced fibrosis than other strains [35]; and the intra-tracheal delivery of bleomycin results in an intense inflammatory reaction within the first week, followed by the development of fibrosis by day 14, with maximal responses at day 21. Therefore, bleomycin administration caused a significant increase in airway stiffness, as judged by the elevation of pressure at airway opening (PAO) in the fibrotic positive controls (bleomycin+Vehicle) compared with the non-fibrotic negative ones (Naïve). The potency of compound **3** was compared with equimolar doses of (±)-ibuprofen and AAZ, given alone or in association, and the results of the present study clearly indicate that compound **3** has shown a sharp anti-inflammatory and anti-fibrotic action, in comparison to positive fibrotic controls (animals treated with Vehicle). The hybrid compound **3** had an increased effect in comparison to (±)-ibuprofen and AAZ given alone or in association.

The late inflammatory response to bleomycin evaluated measuring IL-1β and TNF-α in the supernatant of lung homogenate, demonstrated that bleomycin induces a potent late inflammation, increasing IL-1β and TNF-α production; compound **3** reduced these increases with an effect higher than (±)-ibuprofen and AAZ given alone or in association, confirming its potent anti-inflammatory properties. Moreover, compound **3** stimulates IL-10 production, the most potent anti-inflammatory cytokine involved in resolution of different acute and chronic inflammatory diseases [36].

A link between PGE_2_ production and TGF-β, the most important cytokine involved in the induction of lung fibrosis has been suggested; in fact naproxen down-regulates TGF-β levels and Smad3/4 complex formation [21,37]. Compound **3** was demonstrated to reduce the expression of TGF-ß cytokine, which decreased collagen fiber deposition, as shown in Figure 6A. COX-1/COX-2 inhibitors, such as indomethacin, diclofenac, meloxicam and naproxen, were reported to reduce oxidative stress, inflammation and collagen accumulation in bleomycin-induced lung fibrosis model [22], a model that highlights the inflammatory component of the disease. There are some evidences that PGE_2_ has an anti-inflammatory effect and reduces collagen production [38], suggesting a double role of PGE_2_ in lung fibrosis homeostasis. Our idea is that COX-2 inhibition could have a positive effect on the initial phase of inflammation and a detrimental effect when the fibrotic process is already established.

Carbonic anhydrases are presumably involved in inflammation because this family of enzymes is implicated in pH control by reversibly catalyzing the conversion of CO_2_ to bicarbonate and protons [39]. Recent studies demonstrated positive effects with the use of CAIs in the treatment of inflammatory processes such as arthritis or neuropathic pain [9,40]. Moreover, an unexpected superimposition between anti-inflammatory and CA inhibitory activity was demonstrated with the COX-2 inhibitor celecoxib, opening with this cross reactivity a new scenario for antiglaucoma and anticancer treatments [41].

The results here reported strongly suggest that these new NSAID-CAI hybrid compounds are as promising new anti-inflammatory drugs which could be considered for further development and validation for the treatment of chronic inflammatory diseases such as idiopathic lung fibrosis.

Molecular hybridization is a new concept in drug design and development, based on the combination of pharmacophoric moieties of different bioactive molecules to produce new compounds with improved affinity and efficacy in comparison to the parent drugs [42,43].

This hypothesis is clearly confirmed by our results: in fact, compound **3**, an (±)-ibuprofen fragment linked to a sulfonamide based CAI moiety, has higher anti-inflammatory and anti-fibrotic effects in the mouse model of bleomycin-induced lung fibrosis, in comparison to the reference drugs given alone or in co-administration. Moreover, the ability of compound **3** in inhibiting the production of pro-inflammatory and pro-fibrotic cytokines makes it an innovative anti-inflammatory drug with a dual mode of action and reduced side effects.

The results of the present study give further evidence on the action of these molecules in releasing ache symptoms typical of inflammatory pathologies, such as rheumatoid arthritis [7].

We can affirm that polypharmacology is applicable in different therapeutic areas. New anti-inflammatory drugs developed using molecular hybridization techniques to obtain multiple-ligand drugs can act at multiple targets, allowing for an increased effect and minimizing toxicity. The existing treatment of inflammation is limited by the presence of adverse effects. Combinations of different subunits through the molecular hybridization of anti-inflammatory drugs can create new drugs with better therapeutic activity and better safety profiles [44].

## 5. Conclusions

In conclusion, the results reported here strongly support the notion that these new low molecular weight NSAID-CAI hybrid molecules may act as potential drugs for the treatment of chronic inflammatory diseases such as idiopathic lung fibrosis. Nevertheless, further studies are needed to understand the specific mechanism of action.

## Figures and Tables

**Table 1:** Carbonic anhydrase inhibitory data with compounds **3–6** and acetazolamide (AAZ) as standard against hCA I, II, IX and XII isoforms [7] by a Stopped-Flow CO_2_ Hydrase Assay [27].

Compounds	K_I_ (nM) *			
	hCA I	hCA II	hCA IX ^a^	hCA XII
**3**	48.3	285.0	14.0	6.6
**4**	71.4	47.0	16.3	4.2
**5**	20.9	6.1	8.0	4.1
**6**	93.8	66.4	22.0	5.0
**AAZ**	250	12.1	25.3	5.6

* Mean values from three different assays. Errors were within ±5–10% of the reported values (data not shown). ^a^ Catalytic domain.
